# Color Feature-Based Object Tracking through Particle Swarm Optimization with Improved Inertia Weight

**DOI:** 10.3390/s18041292

**Published:** 2018-04-23

**Authors:** Siqiu Guo, Tao Zhang, Yulong Song, Feng Qian

**Affiliations:** 1 Chinese Academy of Science, Changchun Institute of Optics Fine Mechanics and Physics, 3888 Dongnanhu Road, Changchun 130033, China; guo_qiuqiu@163.com (S.G.); Songyl@ciomp.ac.cn (Y.S.); tsienfung@126.com (F.Q.); 2 University of Chinese Academy of Science, 19 Yuquan Road, Beijing 100049, China

**Keywords:** object tracking, particle swarm optimization, particle maturity, inertia weight, color feature

## Abstract

This paper presents a particle swarm tracking algorithm with improved inertia weight based on color features. The weighted color histogram is used as the target feature to reduce the contribution of target edge pixels in the target feature, which makes the algorithm insensitive to the target non-rigid deformation, scale variation, and rotation. Meanwhile, the influence of partial obstruction on the description of target features is reduced. The particle swarm optimization algorithm can complete the multi-peak search, which can cope well with the object occlusion tracking problem. This means that the target is located precisely where the similarity function appears multi-peak. When the particle swarm optimization algorithm is applied to the object tracking, the inertia weight adjustment mechanism has some limitations. This paper presents an improved method. The concept of particle maturity is introduced to improve the inertia weight adjustment mechanism, which could adjust the inertia weight in time according to the different states of each particle in each generation. Experimental results show that our algorithm achieves state-of-the-art performance in a wide range of scenarios.

## 1. Introduction

In recent years, object tracking technology has been a hot topic in the field of computer vision. For decades, a large number of scholars worldwide have been engaged in the study of object tracking algorithms [1,2,3,4,5,6]. However, object tracking remains a challenging issue due to object complexity, background complexity, and object occlusion. Although there are many sophisticated object tracking algorithms, each algorithm has its own established conditions and application scope. 

The algorithms representative of the traditional object tracking field include the centroid tracking algorithm, the related tracking algorithm, the gate tracking algorithm, the optical flow algorithm, and the mean-shift tracking algorithm. The centroid tracking algorithm and the gate tracking algorithm have satisfactory real-time performance and low complexity, but their stability is poor. In contrast, the related tracking algorithm and the optical flow algorithm have satisfactory stability, but they have difficulty guaranteeing real-time. Compared to the advantages and disadvantages of the previous algorithms, the mean-shift tracking algorithm is the most widely used in engineering. However, the mean-shift tracking algorithm is more susceptible to the background, which is problematic since it is easy to lose the target in a complex environment. In particular, there is no robust algorithm proposed for target occlusion. Most of the solutions are confined to a specific environment and require a large amount of research.

In recent years, many new algorithms have emerged in the field of target tracking. The most popular and representative new algorithms include correlation-filtering-based tracking methods and deep-learning-based tracking methods. Typical algorithms for correlation-filtering-based tracking include the kernelized correlation filter (KCF), the discriminative scale space tracker (DSST) and the spatio-temporal context (STC). The framework of these algorithms is roughly the same. In general, the principle of the correlation filter is that a high (correlation peak) is generated for each interested target and a low response is generated for the background in the scene. In 2010, Bolme proposed a Minimum Output Sum of Squared Error (MOSSE) tracking method based on adaptive correlation filtering in Reference [7]. The algorithm is a pioneering work of introducing correlation filters for visual tracking. In 2015, Henriques proposed a kernelized correlation filter (KCF) which introduced a ridge regression problem with a circulant matrix to apply kernel methods [8]. In 2015, Martin replaced the grayscale features in the MOSSE algorithm with the histogram of oriented gradient (HOG) features that were commonly used in the field of tracking and recognition and proposed a DSST tracking method in Reference [9]. The algorithm relieved the scaling issue using the scale pyramid feature and a discriminative correlation filter. However, because of the complexity of the algorithm, the computational efficiency is low. In 2015, deep learning became very interesting in the tracking field. The use of deep learning could better extract the characteristics of the target and thus better express the target. The algorithm proposed in Reference [10] is an influential algorithm in deep learning. The core idea put forward in Reference [10] was based on the basic tracking framework KCF. The author focused on changing the target characteristics and replacing the HOG features with deep convolution features. The paper proposes learning and expressing the target using the layered convolution feature. They adaptively learn correlation filters on each convolutional layer to encode the target appearance and improve tracking precision. However, the drawback of deep learning lies in the training and speed of the network. Even though the hierarchical convolutional features (HCF) and other offline training are still slow, it is difficult to achieve real-time applications.

Modern tracking algorithms have achieved satisfactory results in the tracking effect, but most are very complex and significantly sacrifice the operation efficiency of the algorithm. For engineering applications, we predict that the algorithm will be simple and easy to use due to its better performance than traditional algorithms and the fact that it can guarantee real-time performance.

In the target tracking system, the selection of target features is related to the tracking effect of the subsequent algorithm. Color features are the most widely used visual features in video tracking. The main reason for this is that color is usually related to objects or scenes contained in images, and when the object rotates and varies in size, the color feature can improve the flexibility of the tracking algorithm. In this paper, the most widely used color histogram is used as the target feature. We have a targeted and reasonable weighting on the histogram to improve the robustness of the algorithm in a complex environment. 

Particle swarm optimization (PSO) has recently been a fast-growing optimization algorithm based on swarm intelligence. The algorithm has good multi-peak search ability, which means it has a good ability to search for a global optimal value and it converges quickly. When the target is occluded during the tracking process, the object similarity function will appear multi-peak. The particle swarm optimization algorithm is used to optimize the object similarity function, which could cope with the target occlusion tracking problem.

In 1995, Dr. Kennedy and Dr. Eberhart proposed a particle swarm optimization algorithm for the optimization of nonlinear functions [11]. The algorithm has the advantages of simple operation and easy implementation, and is widely used in the fields of neural network training, deep learning, and system optimization [12,13,14]. The operation of the algorithm requires some parameters such as inertia weight and the acceleration coefficient. The choice of parameters directly affects the convergence speed and algorithm accuracy. In 1998, Eberhart and Shi proposed a new parameter called inertia weight for the first time [15]. Simulation results showed that this new parameter had a significant and effective impact on the particle swarm optimizer, and when inertia weight was 0.7298, the algorithm had a satisfactory convergence. In 2006, according to the number of iterations, Chatterjee and Siarry proposed a non-linear decreasing adjustment mechanism to adjust the inertia weight [16]. The mechanism allows the inertia weight to be reduced non-linearly from the maximum value to the minimum value. In 2008, Hongguo Zhu presented a random inertia weight adjustment mechanism to improve its global optimization performance [17]. In 2013, Martins Akugbe Arasomwan proposed two adaptive chaotic inertia weight strategies based on the swarm success rate in Reference [18]. In 2013, a new PSO algorithm known as the Gompertz increasing inertia weight (GIIW) was proposed by Wakeel Ahmed, which had satisfactory performance with quick convergence capability [19].

However, the inertial weight adjustment mechanisms proposed in the above paragraph have some limitations, including a constant or random inertia weight adjustment mechanism that is not universally applicable, the linear or non-linear inertia weight adjustment mechanism adjusts inertial weight only by the number of iterations without taking the state of each particle into account, and the adaptive inertia weight adjustment mechanism is generally adjusted by a feedback parameter. However, the feedback parameter cannot be used as a reliable criterion for evaluating inertial weight.

To deal with these problems, this paper introduces the concept of the particle maturity rate to improve the adjustment of inertia weight by taking full account of the different situations of each particle in each generation. The inertial weight is adjusted accurately according to the state of particles to optimize the solution of the object similarity function. The improved particle swarm optimization object tracking algorithm can reduce the number of iterations to achieve the same fitness, which improves the operation efficiency and realizes the tracking of object occlusion.

The main contributions of this paper are summarized below.

(1) We use a weighted color histogram as the object feature description. The object is normalized to a unit circle to reduce the contribution of the object edge pixel in the object feature, which effectively solves the problem of non-rigid deformation, rotation, and scale variation during the object tracking. At the same time, the influence of partial occlusion on the object feature description is reduced.
(2) When the target is blocked in the object tracking process, the object similarity function will reach the “multi-peak” target. Combined with the multi-peak search ability of the particle swarm optimization algorithm, the algorithm is used to optimize the object similarity function to achieve accurate positioning under the object occlusion situation.
(3) An improved particle swarm optimization object tracking algorithm is proposed. The method solves the limitation of the inertia weight adjustment mechanism when the particle swarm optimization algorithm is applied in the field of object tracking.
(4) Experimental results show that the particle swarm optimization object tracking algorithm with improved inertia weight can significantly improve computational efficiency.
(5) Experiments show that the proposed method shows more advantages compared with eleven state-of-the-art algorithms. It is a satisfactory solution to non-rigid deformation, rotation, scale variation, and object occlusion during object tracking.

The rest of the paper is organized as follows. Section 2 introduces the color feature used in the paper and the corresponding similarity measure in detail. The optimized inertia weight adjustment mechanism and the improved particle swarm optimization object tracking algorithm are presented in Section 3. The experimental details of the improved algorithm compared with the eleven most advanced algorithms are discussed in Section 4. In Section 5, we conclude the paper.

## 2. Related Work

### 2.1. Color Feature

Ever since color indexing was proposed by Swain and Ballard in 1991 [20], color features began to be widely used in object tracking. The following is a brief introduction to color feature representation and the corresponding similarity measure used in the paper.

The color feature has less dependence on the size, orientation, and viewing angle of the image, which is why it has higher robustness. Firstly, the image-oriented color feature needs to choose the appropriate color space to describe the color features. Then, we quantify the color features. Finally, we need to define a similarity criterion to measure the color similarity between the images. There are several ways to describe color features, such as histograms, color sets, and color moments. The histogram is not the best nonparametric representation, but it is mostly adequate for use in the tracking process. The histogram is the most widely used color feature in many color tracking fields [21,22,23]. When the image features cannot take all possible values, several zero values will appear in the histogram. These zero values have a large effect on the calculation, which cannot accurately match the two images. In order to solve this problem, we can use the weighted color histogram to represent the color features.

A weighted color histogram of the image region in which the center position of the image is $\left(x_{n},y_{n}\right)$ and the image height and weight are $h_{n}$ and $w_{n}$ is calculated as the image feature. In order to reduce the influence of the target scale, the target is normalized. ${\left\{x_{i}{}^{*}\right\}}_{i=1\ldots N}$ is the pixel coordinate of the normalized initial object. Then, we choose a center convex monotonically decreasing kernel function k(x) as the weight of the pixel to the object center position. We choose the Epanechnikov kernel as kernel function.

(1) $$k(x)=\left\{ \begin{array}{ll} \frac{1}{2}c_{d}^{-1}(d+2)(1-{\left\|x\right\|}^{2}) & \left\|x\right\|\leq 1\\ 0 & otherwise \end{array}\right.,$$

where $c_{d}$ is a constant. $b(x_{i}{}^{*})$ represents the pixel-quantized histogram level. The color histogram quantization level is g, and the object weighted color histogram $M=\left\{M_{j}|j=1\cdots g\right\}$ is represented as Equation (2).

(2) $$M_{j}=C\sum_{i=1}^{N}k({\left\|x^{*}{}_{i}\right\|}^{2})\delta (b(x_{i}{}^{*})-j),$$

where C is the normalization constant. The weighted histogram of the candidate object is defined by the equation below:

(3) $$I_{j}=C_{h}\sum_{i=1}^{N}k({\left\|x^{*}{}_{i}\right\|}^{2})\delta (b(x_{i}{}^{*})-j),$$

where $C_{h}$ is the normalization constant.

The tracking method based on color features has the following advantages.

(1) The amount of the calculation is small. The histogram is a reduced dimension representing the target image, which can reduce the computational complexity of pattern matching.
(2) The histogram generated by a reasonable color model is insensitive to scale variation and rotation. The weighted color histogram also has great advantages for partial occlusion, especially for edge occlusion.
(3) Histogram models are well-suited for modeling non-rigid objects. It is difficult to use shape features to represent a non-rigid object. However, the color distribution is still the same, no matter how the non-rigid object deforms.

### 2.2. Similarity Measurement

A similarity measurement is usually expressed in Hausdorff distance. For two finite point sets $X=\{x_{1},x_{2},\cdots \}$ and $Y=\{y_{1},y_{2},\cdots \}$, the Hausdorff distance between sets X and Y is defined as the equation below:

(4) $$d_{H}(X,Y)=\max\{d(X,Y),d(X,Y)\},$$

where $d(X,Y)=\underset{x\in X}{\max}\;\underset{y\in Y}{\min}\left\|x-y\right\|$ is a distance norm. We define the distance $d_{Y}(x)$ of point x to point set Y as the minimum distance of point y to each point in point set Y. This means $d_{Y}(x)=\underset{y\in Y}{\min}\|x-y\|$. Then, $d_{Y}(X)$ is the distance of each point in point set X to point set Y, and $d_{H}(X,Y)$ is the maximum value of $d_{Y}(X)$. If the known object feature is X and the candidate object feature is Y, then $d_{H}(X,Y)$ can be used to measure the degree of similarity matching between X and Y.

The Hausdorff measurement has different representations depending on the different selected features. The Hausdorff measurement of the gray features mainly includes the mean square error evaluation function (MSE), the sum of absolute difference (SAD), and the normalized cross correlation evaluation function (NC). For the color features, the Bhattacharyya coefficient can be used to express the Hausdorff measurement based on the color histogram.

It is assumed that the normalized color histogram of the template image is $M=\left\{M_{j}|j=1\cdots g\right\}$ and the normalized color histogram of the image to be matched is $I=\left\{I_{j}|j=1\cdots g\right\}$. g is the color quantization level. The Bhattacharyya coefficient indicates the similarity between M and I, which is defined by Equation (5):

(5) $$\rho (M,I)=\sum_{j=1}^{g}\sqrt{M_{j}I_{j}},$$

where ρ(M,I)∈[0,1] and when M is the same to I, ρ(M,I)=1. The larger the value of ρ(M,I), the more similar M is to I.

## 3. Improved Particle Swarm Optimization Method

### 3.1. Particle Swarm Optimization

Particle swarm optimization simulates the predation behavior of birds. It is based on the assumption that a flock of birds randomly searches for food when there is only one piece of food in the area. All of the birds do not know where the food is, but they know how far the current location is from food. Then, the best way to find food is to search for the area around the birds that are currently closest to the food. The particle swarm optimization learns from this model and uses it to solve optimization problems. In particle swarm optimization, the solution to each optimization problem is a bird in the search space, which is called the “particle”. Each particle has a fitness value determined by the objective function. The high fitness value indicates that the obtained solution is accurate. Each particle moves in the solution space whose direction and distance will be determined by a velocity. Then, the particles follow the movement of the current optimal particle in the solution space.

The mathematical equations of the particle swarm optimization algorithm are as follows [24]. In the *n*-dimensional search space, the *i*th particle position is presented as $x_{i}={\left(x_{i1},x_{i2},\cdots x_{in}\right)}^{T}$ and its velocity is presented as $v_{i}={\left(v_{i1},v_{i2},\cdots v_{in}\right)}^{T}$. The individual best particle is presented as $pbest_{i}={\left(pbest_{i1},pbest_{i2},\cdots pbest_{in}\right)}^{T}$ and the population global best particle is presented as $gbest={\left(gbest_{1},gbest_{2},\cdots gbest_{n}\right)}^{T}$. The position and velocity of the particles are updated according to the equations below.

(6) $$v_{id}(t+1)=\omega \times v_{id}(t)+\;c_{1}rand_{1}\left(pbest_{id}(t)-x_{id}(t)\right)+c_{2}rand_{2}\left(gbest_{d}(t)-x_{id}(t)\right),$$

(7) $$x_{id}(t+1)=x_{id}(t)+v_{id}(t+1),$$

where d=1,2,⋯n,i=1,2,⋯PopSize, PopSize is the population size, *c*1 and *c*2 are acceleration constants, $rand_{1}$ and $rand_{2}$ are two distinct random values in [0, 1], *t* denotes the algebra of the current iteration, and ω is the inertia weight. The first part of the equation is the momentum of the particle, which can avoid the swing of the particle in the search direction. The second part of the equation indicates the natural tendency of particles to adapt to the environment. The third part of the equation means information sharing between the particle and neighboring groups [25].

### 3.2. Improved Particle Swarm Optimization Method

The research shows that the particle swarm optimization algorithm has an effective global convergence ability when the inertia weight is large, while the small inertia weight facilitates local convergence. Based on the important influence of inertia weight on the algorithm search behavior, many researchers improve the algorithm optimization performance by changing the inertia weight. At present, the improved method can be divided into a fixed or random inertia weight adjustment mechanism, the linear or non-linear inertia weight adjustment mechanism, and the adaptive inertia weight adjustment mechanism.

**Figure 1:**
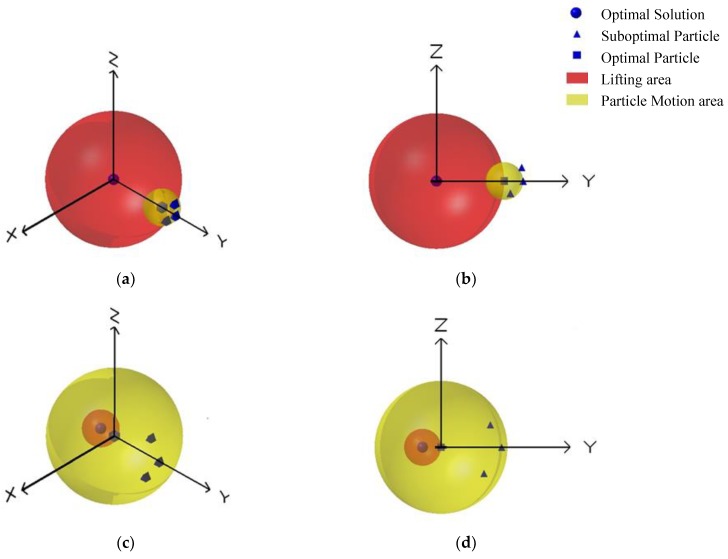
The simplified particle motion model. (**a**) The first case, where the particle swarm is far from the optimal solution, but the particles are close to each other. (**b**) Another perspective of the first case. (**c**) The second case, where the optimal particle is close to the optimal solution and the suboptimal particles also move in the vicinity of the optimal solution. (**d**) Another perspective of the second case.

In order to further explore the effect of inertia weight on particle swarm optimization, the problem is simplified to a unimodal function optimization. We first define that the particle closest to the optimal solution in the particle swarm is the optimal particle and the other particles are suboptimal particles. It should be noted that the fitness value of suboptimal particles may exceed the fitness value of the optimal particle in the process of the particle swarm approaching the optimal solution. At this point, the suboptimal particle that has a better fitness value becomes the optimal particle. This situation constantly happens during the algorithm operation. In Figure 1, the yellow region represents the area where the suboptimal particles may appear next time. The red region indicates that the whole population can obtain a region closer to the optimal solution, which is called the lifting area. The closer the particle is to the optimal solution, the higher the fitness is. It is obvious that the size of the lifting region is determined by the distance of the optimal particle to the optimal solution. 

For the first case shown in Figure 1a, the particle swarm is far from the optimal solution, but the particles are close to each other. When the particles are far from the optimal solution, it is necessary to iterate several times to get closer to the optimal solution. In this case, since the optimal particle needs a large velocity to approach the optimal solution, the value of inertia weight should be relatively large. The velocities of suboptimal particles are determined by their previous velocity and their distance to the optimal particle. Therefore, the suboptimal particles also need a large inertia weight value.

For the second case shown in Figure 1c, the optimal particle is close to the optimal solution and the suboptimal particles also move in the vicinity of the optimal solution. When the suboptimal particles approach the optimal solution at a high speed, they will rush though the lifting area and continue to oscillate in the yellow area, which means the particles will not enter the lifting area. In this case, in order to improve the accuracy of the solution, it is necessary to reduce the velocities of the particles, which means the particles will require a smaller inertia weight value to approach the optimal solution slowly.

Both of these situations may occur during the algorithm operation. Fixed or random inertia weight adjustment mechanisms are not appropriate. The method of adjusting the inertia weight linearly or non-linearly by the number of particle iterations is to use a moderate inertia weight to make the optimization achieve satisfactory results without taking full account of the different situations of each particle in each generation. The adaptive inertia weight adjustment mechanism adjusts the inertia weight according to the feedback parameter. Usually, the feedback parameter will use the fitness value or its derived value. However, the fitness value is not a reliable standard for evaluating inertia weights. When the particle has a high fitness value, it may require different values of inertia weight to adjust because of the different situations of the particle. When the fitness value of the particle is low, it may have different requirements for inertia weight.

In order to solve the above problems, this paper presents an improved inertial weight adjustment mechanism, which introduces the concept of the particle maturity rate as a description of particle states. When the particle maturity rate is relatively high, it indicates that the particles are clustered in a position far from the optimal solution and the whole population is slowly approaching the optimal solution. This is shown in Figure 1a. When the particle maturity rate is relatively low, it shows that the particles oscillate near the optimal solution. This is shown in Figure 1c.

First of all, we define coefficient $\sigma_{i}(t)$ as a value that can indicate the speed of particle maturity. The large coefficient value indicates that the particle matures quickly. $\sigma_{\max}(t)$ is the maximum value of $\sigma_{i}(t),i=1\ldots Popsize$.

(8) $$\sigma_{i}\left(t\right)=\frac{1-pbest_{i}(t-1)}{1-pbest_{i}(t)}$$

The maturity rate of the *i*th particle at *t*th iteration is defined by the equation below:

(9) $$M_{i}\left(t\right)=\left\{ \begin{array}{ll} \frac{1-pbest_{i}(t-1)}{\sigma_{\max}(t)-pbest_{i}(t)\times \sigma_{\max}(t)} & \sigma_{i}(t)>1\\ {\left(\frac{pbest_{i}(t)\times \sigma_{i}(t)}{2}\right)}^{2} & \sigma_{i}(t)\leq 1 \end{array}\right.,$$

where $pbest_{i}(t)$ is the individual optimal solution found by the *i*th particle at the *t*th iteration. Combined with the particle maturity rate formula, the maturity of the entire population can be described by Equation (10):

(10) $$S_{m}\left(t\right)=\frac{\sum_{i=1}^{PopSize}M_{i}\left(t\right)}{PopSize}.$$

$S_{m}(t)\in \left[0,1\right]$ represents the maturity rate of the entire population. When $S_{m}(t)$ is small, it indicates that the particles are mostly in the first situation and, when $S_{m}(t)$ is large, it shows that most of the particles are in the second situation. Using $S_{m}(t)$ to adjust inertia weight, the status of each particle in each generation can be used as a basis for adjustment. In this paper, $S_{m}(t)$ is used to adjust inertia weight linearly and the formula is shown below:

(11) $$\omega \left(t\right)=\omega_{\min}+S_{m}(t)(\omega_{max}-\omega_{min}).$$

The inertia weight varies between the maximum and the minimum. $\omega_{\max}$ and $\omega_{\min}$ are selected to be [0.2, 0.8] according to experience. Because $S_{m}(t)\in \left[0,1\right]$, the range of inertia weight is reasonable.

### 3.3. Improved PSO Object Tracking Method

In this paper, the improved particle swarm optimization object tracking method is combined with the concept of the particle maturity rate to calculate the maturity rate of the entire population by taking into full account the situation of each particle in each generation. The maturity rate of the entire population is used to adjust the inertia weight, which means the adjustment of the inertia weight is more reasonable and the algorithm efficiency is improved. The main steps of the algorithm are provided in Algorithm 1.

**Algorithm 1.** Improved particle swarm optimization object tracking method1: Initial parameters of the particle swarm. Initial $v_{i}\;\mathrm{and}\;x_{i}.$2: Initialize the target box in the first frame. Determine the object feature description and similarity measure.3: **For** frame = 2, 3, ... to the last frame.4: **For**
*t* = 1, 2, ... to the maximum number of iterations.5: Calculate the $v_{i}$ according to Equation (1).6: Calculate the $x_{i}$ according to Equation (2).7: **If** fitness value of the current particle is better compared to individual optimal solution.8: Update the individual optimal solution $pbest_{i}=x_{i}$.9: **End.**10: **If** fitness value of the current particle is better compared to global optimal solution.11: Update global optimal solution, $pbest_{i}=x_{i}$.12: **End.**13: Adjust inertia weight according to Equation (6).14: **End.**15: Locate the target by using the maximum fitness value position.16: **End.**

## 4. Experimental Results and Analysis

### 4.1. Object Tracking Effect

We performed the experiments on a PC with Intel i5-5200U CPU (2.2 GHz), 8 GB RAM memory, and Windows 7 operating system. The experiments in this paper were implemented in MATLAB 2016. We compared the performance of the improved algorithm with eleven state-of-the-art algorithms including TLD [26], Struck [27], VTD [28], CSK [29], CT [30], DFT [31], OAB [32], MIL [33], KCF [8], DSSL [9], and DLSSVM [34]. The population size in our algorithm was set to 100, the maximum number of iterations was 180, and the parameters were fixed during the whole experiment. For the other eleven algorithms in this paper, we used the code that they have already provided, including the best parameter values. We tested all relevant video sequences in OTB2013 and OTB2015, and some representative tracking results are shown as follows.

#### 4.1.1. Non-Rigid Deformation

**Figure 2:**
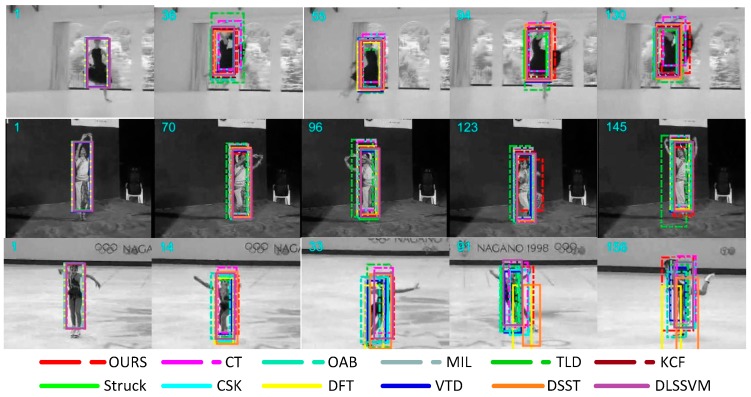
Tracking results of the proposed method and eleven state-of-the-art tracking methods on sequences *Dancer*, *Dancer2*, and *Skater*. In these sequences, there is a non-rigid deformation problem.

For the non-rigid deformation in the object tracking process, *Dancer*, *Dancer2*, and *Skater* were used as the representative test sequences, and the partial tracking results are shown in Figure 2. In the *Dancer* sequence, the TLD algorithm began to lose the target after the target was deformed for a period of time, which would not accurately track the target. In the *Dancer2* sequence, when the object was non-rigidly deformed for a period of time, the TLD and CT algorithms showed tracking frame drift. In the *Skater* sequence, DFT and DSST algorithms failed to track the target after a period of time after target deformation. During the whole tracking process, our algorithm was more accurate and stable. The image single frame processing speed was better than 23 milliseconds. This can be attributed to the use of color features, which are not sensitive to appearance deformation.

#### 4.1.2. Scale Variation

**Figure 3:**
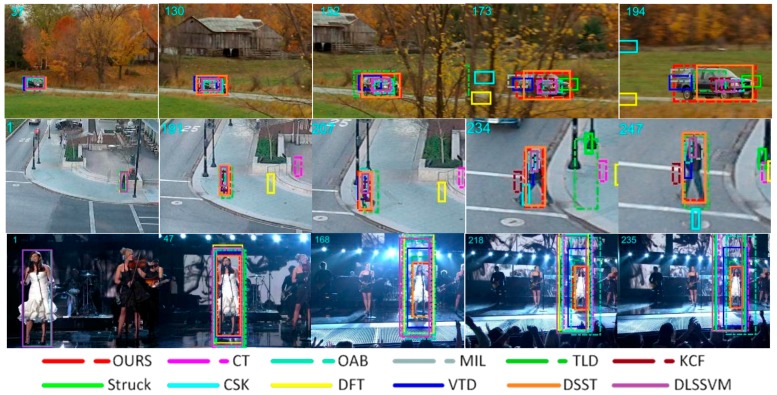
Tracking results of the proposed method and eleven state-of-the-art tracking methods on sequences *Carscale*, *Human6*, and *Singer1*. In these sequences, there is a scale variation problem.

In Figure 3, *Carscale*, *Human6*, and *Singer1* were used as the test sequences with scale variation during the object movement. In the *Carscale* sequence, there are not only obvious changes in scale, but there is partial occlusion. In the 152nd frame, when the target size varies greatly, the algorithm proposed in this paper started to show clear advantages. In the *Human6* sequence, DFT and CT algorithms began to lose the object from the 191nd frame. In subsequent image sequences, the target scale changes more significantly and the proposed algorithm could always track the target steadily. In the *Singer1* sequence, our algorithm and DSST algorithm showed satisfactory tracking performance compared to other algorithms. Throughout the tracking process, our tracking window could mostly contain the object and the single frame image processing speed was better than 36 milliseconds.

#### 4.1.3. Rotation

**Figure 4:**
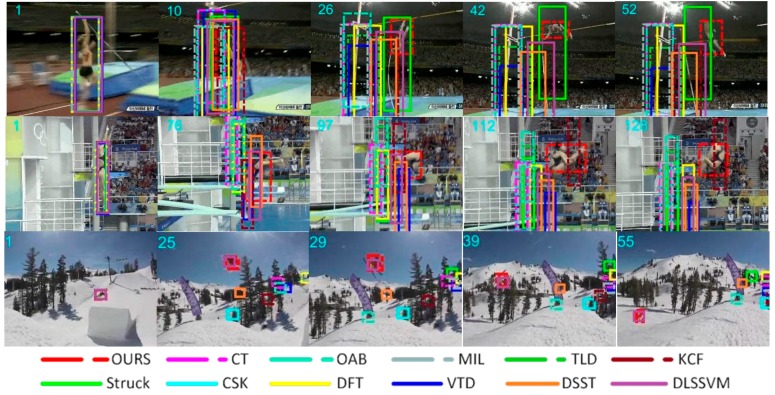
Tracking results of the proposed method and eleven state-of-the-art tracking methods on sequences *Jump*, *Diving*, and *Skiing*. In these sequences, there is a rotation problem.

Figure 4 shows the tracking results with rotation. *Jump*, *Diving*, and *Skiing* are classic target rotation sequences, and were used as the test image sequences. In the *Jump* sequence, the target starts rotating from frame 10 and the algorithm proposed in this paper could solve the rotation problem better than other algorithms. In the *Diving* sequence, starting from frame 76, other algorithms began to lose the target, in addition to the proposed algorithm. In the *Skiing* sequence, our algorithm and the DLSSVM algorithm showed good performance and achieved a stable tracking target in fast rotation. In the entire process of tracking, the proposed algorithm could stably lock the target. In this scenario, the single frame image processing speed was better than 40 milliseconds.

#### 4.1.4. Occlusion

**Figure 5:**
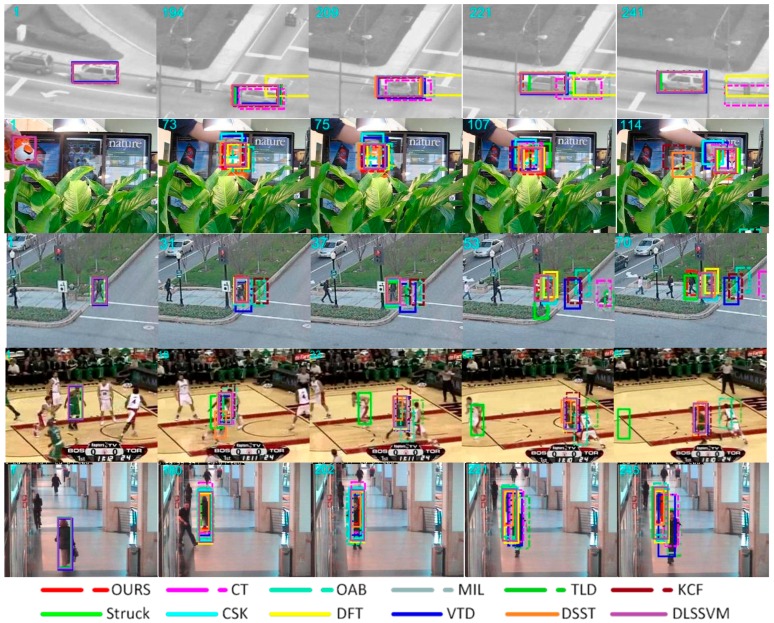
Tracking results of the proposed method and eleven state-of-the-art tracking methods on sequences *Suv*, *Tiger2*, *Human3*, *Basketball*, and *Walking2*. In these sequences, the objects undergo occlusion.

In order to fully reflect the advantage of the algorithm proposed in this paper in the case of the similarity function occurring at “multi-peak”, we used *Suv*, *Tiger2*, *Human3*, *Basketball*, and *Walking2* as test sequences to track the occluded object. The tracking results are shown in Figure 5. In the *Suv* sequence, CT and DFT algorithms lost the target when the target passed through the blocking card while the tracking algorithm proposed in this paper could accurately and stably track the target. In the *Tiger2* sequence, the object was repeatedly obscured by the green leaves and most of the tracking algorithms deviated from the object. The tracking algorithm proposed in this paper has obvious advantages, and the tracking box covered the object position accurately. In the *Human3* sequence, the object is blocked completely for a short period of time. Starting from frame 53, tracking algorithms such as VTD, TLD, and Struck—which are traditionally well-behaved when dealing with occlusion—started to lose the object. However, the tracking algorithm proposed in this paper still stably locked the object. When the object appeared again in the image, the proposed tracking algorithm immediately identified the object to achieve the robustness of the entire tracking process. In the *Basketball* sequence, the object is heavily or even completely blocked. The algorithm proposed in this paper had good performance in managing the occlusion problem. In the *Walking2* sequence, when the target is out of occlusion, only a few algorithms (e.g., our algorithm and the DLSSVM algorithm) could continue to track the target. It can be seen that the improved particle swarm optimization tracking algorithm has an effective anti-occlusion ability, which basically guarantees the real-time value.

### 4.2. Algorithm Efficiency Comparison

In order to show that the improved particle swarm optimization object tracking method proposed in this paper has fewer iterations and higher tracking efficiency than other particle swarm optimization object tracking methods, the five sets of image sequences in the previous section were compared experimentally using the nonlinear inertia weight adjustment mechanism, the adaptive inertia weight adjustment mechanism, and the improved inertial weight adjustment mechanism, respectively. Because the linear and nonlinear inertia weight adjustment mechanisms are relatively similar, this paper uses the non-linear adjustment mechanism as its representative. We applied different PSO weight adjustment mechanisms to complete 20 experiments for each group of image sequences and recorded the experimental data to calculate the average single frame. When the object similarity function value reached 0.98, the results are shown in Table 1.

Method 1 is a non-linear decreasing inertia weight adjustment method. Method 2 is an adaptive method that dynamically changes the value of the inertia weight. Method 3 is the improved inertial weight adjustment method proposed in this paper.

As can be seen from Table 1, for the *Suv* sequence, the three methods could basically guarantee robustness and real-time due to less obstruction. However, the computational efficiency of the improved particle swarm tracking algorithm was clearly higher than the nonlinear method and the adaptive method. For *Tiger2* sequences, the computational efficiency of Method 1 and Method 2 were significantly reduced because of the more serious occlusion of image sequences. However, the improved method proposed in this paper could basically guarantee real-time tracking under partial occlusion, showing that the improved method has greatly enhanced algorithm efficiency. For *Human3*, *Basketball*, and *Walking2* sequences, the efficiency of the improved method was obviously better than the non-linear method. Compared with the adaptive method, the improved method significantly reduced the number of iterations when the object was severely obstructed, and it improved the efficiency of the algorithm. Experimental results showed that the improved algorithm could accurately meet the algorithm requirements by adjusting the inertia weight according to the different states of each particle in each generation, which can effectively improve the computational efficiency of the algorithm.

### 4.3. Algorithm Effect Comparison

In this subsection, we evaluate the algorithm performance with one-pass evaluation (OPE), which is proposed in Reference [35]. We used two assessment criteria, including the success and precision plots. The precision plots are defined as the percentage of frames with the center position error less than the predefined threshold. The success plots represent the percentage of the frames in which the overlap rates of the tracking area and the boundary frame are larger than the threshold. For precision plots, the results at the error threshold of 20 were used for ranking. The area under the curve (AUC) of each success plot was used to rank the tracking algorithms.

**Figure 6:**
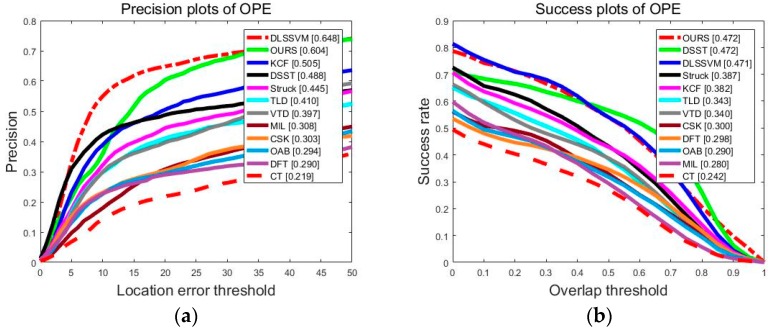
(**a**) Precision plots and (**b**) Success plots of one-pass evaluation (OPE) for 12 trackers. The legend reports the performance score for each tracker.

Figure 6 shows the overall performance of the twelve trackers. It can be seen that our algorithm ranked first in success plots. In precision plots, our algorithm ranked second, which is only 4.4% worse than the first DLSSVM algorithm. The precision of our algorithm was improved by 9.9% compared to the top-level KCF algorithm in the precision plots. In the success plots, our algorithm achieved an AUC of 0.472 and the performance of our algorithm was equal to the top-ranked DSST algorithm in the related filtering algorithm. The precision and success plots show that our algorithm had superior tracking performance compared to 11 advanced tracking algorithms in terms of the video sequences in OTB2013 and OTB2015.

**Figure 7:**
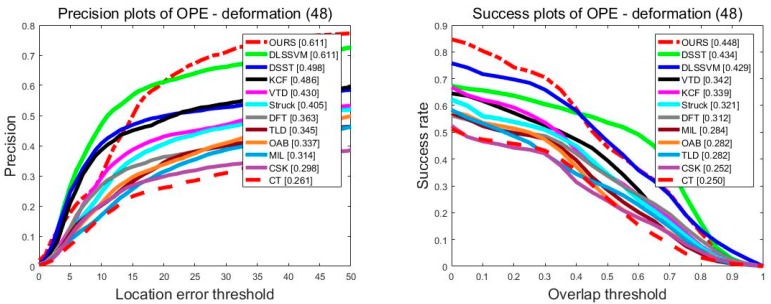

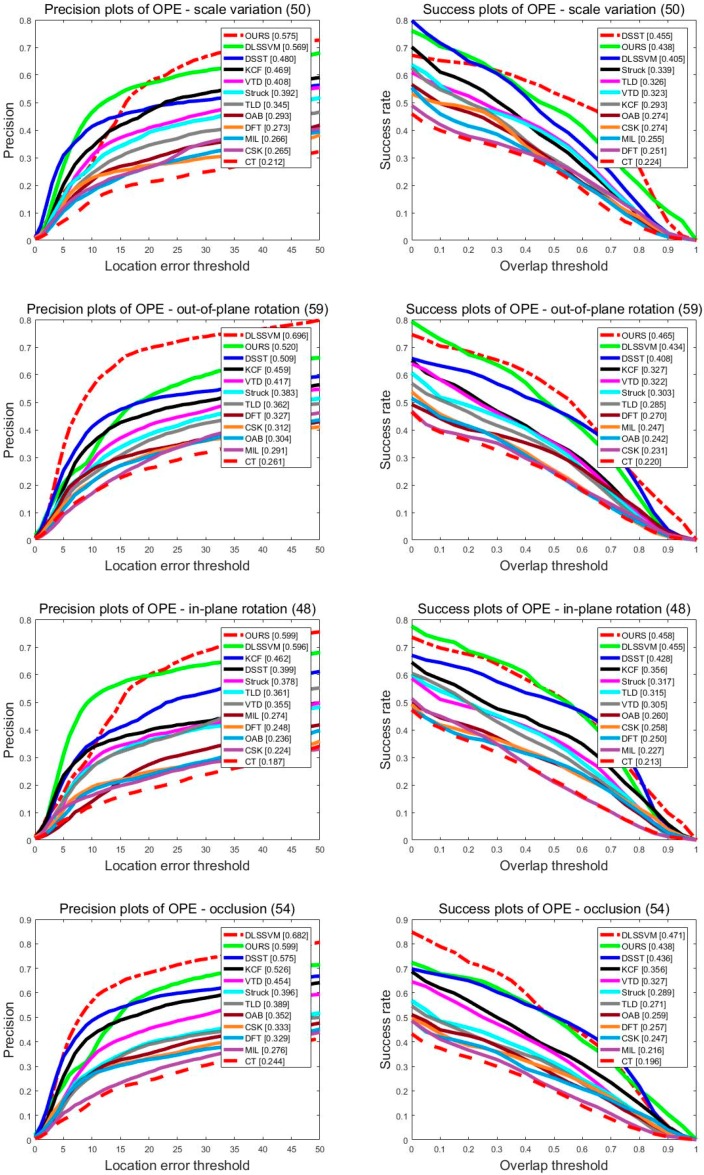
The precision plots and the success plots of sequences with non-rigid deformation, scale variation, rotation, and occlusion. The performance score of each tracker is shown in the figure.

Figure 7 analyzes the performance of the twelve trackers in various tracking states. For all test video sequences, there were 48 groups belonging to object deformation, 50 groups belonging to object scale variation, 59 groups belonging to the out-of-plane rotation group, 48 groups belonging to the in-plane rotation group, and 54 groups belonging to object occlusion. It should be noted that one group of the image sequence may belong to different kinds of groups at the same time because the group of image sequences may appear in a variety of situations. For example, there is not only scale variation, but also a partial occlusion in the *Carscale* sequence. Figure 7 shows the precision plots and success plots, respectively, in various situations. Our algorithm stands out in every situation. For the deformation image sequence, our method ranked first in precision plots and success plots, and was significantly better than other methods. In the scale variation image sequence, our method ranked first, with 0.575 in precision plots. In success plots, our algorithm was only 1.7% worse than the DSSL algorithm, which achieved an AUC of 0.438. In the rotation image sequence, whether it is out-of-plane rotation or in-plane rotation, our algorithm performed significantly better than DSST, TLD, KCF, and other methods. In addition to out-of-plane rotation, the DLSSVM algorithm took first place, with an advantage of 1.7%. In the occlusion image sequence, our algorithms ranked after the DLSSVM algorithm both in precision plots and success plots. However, our algorithm still achieved a good score of 0.599 in precision plots. In success plots, our algorithm achieved an AUC of 0.438, which is obviously better than the other ten algorithms such as the DSST and KCF.

It can be seen that the particle swarm tracking algorithm with a color histogram feature has clear advantages in dealing with non-rigid deformation and rotation. This may be due to the fact that the weighted color histogram feature is insensitive to deformation and rotation. Our algorithm also had good performance in dealing with scale variation. However, our algorithm ranked after the DSST algorithm in success plots. As described in Section 1, the DSST algorithm is specifically designed to make effective improvements to scale variation, and thus has excellent performance in dealing with scale variation. Although our algorithm ranked behind the DSST algorithm, it is still superior to other advanced algorithms when dealing with scale variation issues. For the case of occlusion, the DLSSVM algorithm performed better than our algorithm in tracking effects. The DLSSVM algorithm is an improved machine-learning-based tracking algorithm, and has better performance than traditional machine-learning-based algorithms. However, the tracked structure is still complex. The target tracking is achieved through the cooperation of the detector and tracker. A large number of target samples are learned by using the classifier to improve the detection effect. When dealing with occlusion issues, the machine-learning-based tracking algorithm has significant advantages. Our algorithm uses the simplest tracking structure. Particle swarm optimization is used to optimize the target similarity function to achieve a sufficient tracking effect. By improving the inertia weight adjustment mechanism, we improved the efficiency of the algorithm’s operation. Therefore, compared with the DLSSVM algorithm with complex structure, our algorithm had difficulty surpassing the tracking effect, but it had clear advantages in tracking efficiency. Compared to the other ten algorithms, when the object was blocked, the improved particle swarm tracking algorithm could still fully exploit its own characteristics in dealing with the various occlusion situations, and could stably lock the object.

## 5. Conclusions

This paper presents a new object tracking method. A weighted color histogram normalized to a unit circle is used as the target feature description. Particle swarm optimization is used to optimize the object similarity function. When the target undergoes occlusion, the object similarity function will appear at the multi-peak situation and the multi-peak search ability of the particle swarm optimization algorithm is utilized to realize the accurate positioning in the occlusion situation. When the particle swarm optimization algorithm is applied to object tracking, the inertia weight adjustment mechanism has some limitations, and we present an improved method. We introduce the concept of particle maturity to achieve efficient optimization. The algorithm can adjust the inertia weight in time according to different conditions of each particle in each generation and greatly reduce the amount of computation. The experimental results show that the improved particle swarm optimization object tracking method achieves the same fitness with a smaller number of iterations. The proposed algorithm shows the most advanced performance in a wide range of scenarios.

## Figures and Tables

**Table 1:** Algorithm time-consuming contrast (ms).

Attributes	Method 1	Method 2	Method 3
*Suv*	65	38	3
*Tiger2*	140	102	26
*Human3*	390	252	35
*Basketball*	510	430	42
*Walking2*	367	222	39
